# Traffic police officers’ experience of post-crash care to road traffic injury victims: a qualitative study in Tanzania”

**DOI:** 10.1186/s12873-019-0274-x

**Published:** 2019-10-11

**Authors:** Gift G. Lukumay, Anne H. Outwater, Dickson A. Mkoka, Menti L. Ndile, Britt-Inger Saveman

**Affiliations:** 1 Department of Community Health Nursing, Muhimbili University of Health and Allied Sciences (MUHAS), Dar es Salaam, Tanzania; 2 Department of Clinical Nursing, MUHAS, Dar es Salaam, Tanzania; 3 Department of Nursing, Umeå University, Umeå, Sweden

**Keywords:** Post-crash care experience, Qualitative method, Road traffic injury, Traffic police

## Abstract

**Background:**

Recently, road traffic injuries (RTIs) have become a major health problem affecting health systems in many low- and middle-income countries. Regardless of whether an ambulance is available for evacuation, police officers have been shown to arrive at the crash scene first, becoming, in effect, the first responders to RTI victims. Therefore, the study aimed to explore the experiences of traffic police officers in regard to the provision of care to RTI victims in the prehospital environment, including the role of traffic police upon arriving at the crash scene, the challenges they face, and their opinions about how to improve care to RTI victims.

**Method:**

The study used a qualitative approach in which data were obtained from 10 individual interviews and three focus group discussions. There were 41 participants, 27 of them were male and 14 were female. About half (48.7%) of the study participants were aged between 30 to 39 years. Qualitative content analysis was used to analyse all the materials.

**Results:**

Three themes emerged from the analysis. The theme “Maintain safety while saving injured victims’ lives and facilitating access to a health facility” was comprised of safety, sorting, initial help, and assisting access to hospital care. “Overwhelmed working with limited resources and support” included limited care and transport resources, police fatigue, and little or no support. “Improving supportive system and empowering frontline personnel” included the need for an emergency care system, availability of resources and an emergency medical support system, and training for police and drivers regarding victims’ first-aid care, and road safety.

**Conclusion:**

The study findings characterize an environment in which the police first responders have no knowledge or skills and no equipment and supplies to provide care to RTI victims at the scene before rushing them to definitive care. The results suggest a favorable climate for training and equipping officers so that they can deliver competent postcrash care at the scene while emergency medical services are yet to be established. However, more research will be needed to determine the efficacy of such training and its acceptability in the Tanzanian context.

## Background

Road traffic injuries (RTIs) have become a major health problem in many low- and middle-income countries (LMICs) [1]. RTI is the leading cause of injury death, worldwide, among those ages 15–29 years [2, 3]. This is in part because road transport is the main mode of transport worldwide [4]. In Tanzania, the site of the present study, road transport accounts for three quarters of all types of transport [5]. Because most economic activities involve movement from one place to another, people in the economically active age groups have increased exposure to RTIs.

The impact of RTIs on economic development is substantial. The increased number of deaths and disabilities among people of reproductive age has become a global public concern [1, 6–8]. Disabilities from injuries always interfere with victims’ economic activities and affects their overall quality of life [9]. Furthermore, victims who survive often require long-term health care if they are to recover. Hence, RTIs increase the burden on the health care system, which in many countries is already overwhelmed by other health problems [10]. As well, the families of RTI victims can become impoverished by the need to expend increased resources to cover treatment costs. The World Health Organization (WHO) has found that on a global basis, about 5% of gross domestic product (GDP) is spent on goods and services related to RTIs [2]. However, it is now agreed that the impact of RTIs can be minimized if post-crash care is maximized [11].

Effective post-crash care is supposed to include prompt communication with and activation of the emergency medical system (EMS), if one is available; a prompt response from the activated system; and assessment and treatment of the victim on site, followed by expeditious transport to an appropriate health facility [12]. The care that RTI victims receive from prehospital providers ranges from no care at all to high-quality care, depending on the knowledge and skills of the providers. Because a minute can make a difference between life and death in the prehospital environment, intervention should be appropriate for the best outcome. Studies show that interventions range from basic life support (BLS) to advanced life support (ALS) [13]; although ALS includes more components, it has been shown to be complicated, to be an inefficient use of resources, and to be no better than BLS measures at saving lives at the crash scene [14].

While most high-income countries (HICs) have an advanced EMS [15], few LICs have any system-level form of emergency medical services; further, it has been found that even where such systems exist in LICs, 98% of individuals who use them are not satisfied with the care providers [16]. Tanzania is one of the great majorities of LICs where an EMS is not available. Hence, in most cases, RTI victims are transported to the hospital by untrained first responders who, at best, can provide little initial care to stabilize their condition [17, 18].

Regardless of whether an ambulance is available for evacuation, police officers have been shown to arrive at the crash scene first, becoming, in effect, the first responders to RTI victims [19]. Other key responders commonly involved in evacuating and transporting RTI victims to the hospitals are relatives and taxi and motorcycle drivers [20]. However, in most cases police become involved in post-crash care directly or indirectly by providing instructions to other responders and coordinating transport for RTI victims. Despite the role of the police as key first responders in post-crash care, evidence shows poor outcomes for victims who receive direct physical care from police before reaching the hospital [21]. The findings of a recent study show that these poor outcomes are due to poor knowledge about post-crash care provision and poor post-crash care skills [22]. Although the study found that 99.1% of officers had a positive attitude toward caring for RTI victims, the officers reported performing many incorrect interventions during immediate post-crash care that could endanger victims’ lives.

Regarding prehospital care in Tanzania, at least one study has focused on the prehospital experiences of injured victim [23], yet little is known about the experience of first responders in providing initial post-crash care to victims of RTIs. Therefore, in the present study we aimed to explore the experiences of traffic police officers in regard to the provision of care to RTI victims in the prehospital environment, including the role of traffic police upon arriving at the crash scene, the challenges they face, and their opinions about how to improve care to RTI victims.

## Method

### Study design

We selected a qualitative explorative study design in which we used individual interviews and focus group discussions (FGDs) with traffic police officers in Dar es Salaam Region, Tanzania.

### Setting

The present study was conducted in Tanzania’s Dar es Salaam Region, which has an area of 1590 km^2^. The region consists of three districts: Ilala, Temeke, and Kinondoni. The city of Dar es Salaam is a major commercial seaport and Tanzania’s largest city, with an estimated population of more than 5.7 million [24]. Dar es Salaam Region, which is coextensive with the city of Dar es Salaam, was selected as the setting for the present study because, according to a 2015 report by the national government, it had the nation’s highest number of road traffic incidents, accounting for more than a third of all such incidents [25]. Also, traffic police in the city have been shown to transport RTI victims to the hospital by using police patrol cars or private cars. However, it has been found that more than 50% of victims transported by the police die before they arrive at the hospital [21]. While we could have conducted a study in any jurisdiction in Tanzania, Dar es Salaam is the only region in the country with a designated public trauma hospital: Muhimbili Orthopaedic Institute.

### Sampling methods and data collection

Using a designed interview guide, we conducted individual interviews and FGDs in three phases from December 2017 to June 2018. In the first phase (December 2017), initial exploratory individual interviews were conducted with four traffic police officers about their experiences with post-crash care. These interviews resulted in the emergence of issues related to the officers’ responsibilities, challenges they faced, and ways, going forward, to reduce challenges and provide better care that warranted further exploration. In the second phase (March–April 2018), six more traffic police officers were individually interviewed. During phase three (May–June 2018), three FGDs were conducted; two FGDs had 10 participants while the third had 11 participants. Participants were selected from traffic police officers who had participated in a recent survey study [22]. Those traffic police officers who said they had cared for more than six trauma patients in the previous year were selected as key informants for the study. To ensure maximum variation, we included 10 police constables, eight corporals, 11 sergeants, seven assistant inspectors of police, and five inspectors of police.

Before data collection, the researcher briefed the selected traffic police officers about the study. The officers were also told that the study had received an ethical clearance from the MUHAS Senate Research and Publication Committee. Permission to interview traffic police officers was granted by the Office of the Inspector General of Police (IGP), Tanzanian Ministry of Home Affairs, after submission of the MUHAS letter of ethical clearance. Lastly, the officers were informed that participation was voluntary. From the response the researcher received, all traffic police officers who were selected and invited wanted to be part of the study. Before the interview started with each traffic police officer selected for the study, written informed consent was obtained.

Data were collected and stored on a password-protected laptop computer. One in-depth interview or one FGD was conducted per day lasting 40–50 min. All interviews were tape-recorded.

### Data analysis

Content analysis was used to analyse the data [26]. The first author started by listening to the audiotaped interviews and transcribing them verbally before translating them from Swahili to English. The transcripts were analysed by reading and re-reading them to become familiar with the data. Transcripts from individual traffic police interviews and FGDs were analysed for identification of meaning units related to informants’ perspectives on experiences during the provision of post-crash care. Further, meaning units relating to the officers’ role when it came to helping RTI victims, challenges the officers faced when fulfilling their role, and their opinions on how to approach those challenges were also identified. Then, codes were extracted from these condensed-meaning units. The codes from the text were further analysed in order to distinguish similarities and differences. Then, similar codes were grouped together to form categories reflecting the manifest content of the text. Lastly, the text and the categories were read again to capture their underlying meaning.

## Results

There were 41 traffic police officer, working from three districts in Dar es Salaam region who were interviewed for this study. The mean age of the participants was 37 years. Participants differed in terms of sex/gender where by 27 where men and 14 women. The working experience as a traffic police officer ranging from 3 year to 32 years, while about half (48.7%) of them aged between 30 and 39 years old.

It was found that traffic police officers are the first responders on the crash scene in most situations; as they step onto the scene, they experience the role of rescuer as well as that of a traffic police officer. A strong inner fear of touching individuals who are injured arises, the officers said, but they reported that they find courage, and calm themselves in order to fulfil their duties of clearing the scene, maintaining safety (their primary role), and meeting others’ expectations of transporting injured victims to the hospital and providing some initial help when possible. Doing work that is not easy even for professional rescuers, the officers are further challenged by limited access to training and technical equipment; this creates a difficult environment that puts their lives and those of the victims who receive care from them at increased risk. With the aim of reducing these challenges, traffic police officers raised suggestions about possible ways of reducing the challenges and ensuring that they would be safer while managing a crash scene. From analysis of descriptions of initial post-crash care of RTI victims from the injury scene to the hospital, three themes emerged: *Maintaining safety while saving injured victims’ lives and facilitating access to the health facility* refers to the officers’ perceived role of maintaining personal, bystander, and victim safety, as well as helping victims all the way from the scene to the hospital. *Overwhelmed while working with limited resources and support* illustrates how the traffic officers face challenges in fulfilling their role of providing post-crash care. *Improving support system and empowering frontline personnel* refers to the officers’ views on actions that should be taken to support their role of providing initial post-crash care of RTI victims (Table 1).

**Table 1:** Themes, categories and selected codes that emerged during analysis

Theme	Category	Selected codes
Maintain safety while saving injured victims’ lives and facilitate access to health facility	Providing initial help to injured victim	Remove victim from scene
		Positioning victim
		Fan serious victims
		Provide first aid if possible
	Sorting out victims while ensuring safety	Cordon the scene.
		Prevent further injuries.
		Identfy surviving victims and the dead
		Notify citizen about incident
	Transporting RTI victims to the hospital	Rush to hospital using police car
		Request a private car for transport
		Forcing car owner to rush victim to hospital
		Paying car owner to rush victim to hospital
Overwhelmed while working with limited resources and support	Working with scarce resources for helping victims	No first-aid kits and stretchers.
		Use cloths to prevent blood contact
		Use plastic bags
		Caring for victim with bare hands
	Difficulty facilitating victims’ access to health facilities for care	Use personal cars.
		Private car owners’ resistance to carrying victims.
		Unavailability of ambulances at scene.
		Bad roads.
	Overwhelmed and exhausted with responsibilities without resting	Heavy workload with little resting time
		Working overtime over phone while at home
		Becoming stressed while working with victim
		Helping but being blamed by civilians
	Lacking support system at the scene and at health facilities	Overcrowding at the scene with no help
		Onlooker stealing victims’ property
		Bureaucracy delays admission of patient to emergency department
		Delays in receiving victim/dead body at emergency department
Improving the support system and empowering frontline personnel	Need for strong emergency care system	Available standby ambulance along major roads
		Special number to call ambulances
		Special emergency number to call hospital
		Preparedness of emergency care provider
	Availability of resources and emergency medical support system	Distribution of first-aid material
		Increased number of police patrol cars
		Specified police patrol car for victim
		Employing more staff in police posts
	Training for police and drivers on victims’ first aid and road safety	First-aid training for police and other drivers
		Training on road rules and regulations for drivers
		Workshop on care for different types of injured patients
		Not being rough while caring for the victim

### Theme 1: maintaining safety while saving injured victims’ lives and facilitating access to the health facility

The traffic police officers said that in addition to their primary role of maintaining safety, they also provided some initial management and helped injured victims reach the health facility for proper care.

#### Providing initial help to the injured victim

The participants reported several initial actions they took to help RTI victims. Once they arrive at the scene, the first thing the police officers do is to remove the injured victim(s) from the vehicle and crash scene and place them out of harm’s way under a shady tree or at the side of the road. Although it was described that participants placed the victims on their back, the officers could not explain clearly why they did so. However, one participant described that this position helps the victim to breathe, and in case of respiratory difficulty the victim’s face can be fanned easily to increase ventilation. Despite mentioning the provision of first aid to victims, the participants were not clear about what type of first aid they normally provided. They further noted that they did not know what to do for first aid; most of time they rushed victims to the hospital for such services.

“I am supposed to provide first aid to injured victims, but it always depends because you might be busy doing something to a victim, thinking that you are helping; instead, you are worsening the victim’s condition..... That is why I always rush them to the hospital, which I believe is a safe place for them”. (FGD2, Participant 4)

#### Sorting out victims while ensuring safety

Apart from providing initial help to injured victims, participants reported that they ensured safety at the crash scene by cordoning off the scene area to alert other road users that there had been a crash and that they should not themselves become victims (or worsen the state of already-injured persons) by intruding onto the scene, whether in a vehicle or on foot. Personal safety was mentioned as the top priority before helping the victims and the main reason they cordoned off the scene. Participants also reported that upon arrival at the scene they sorted out victims, identifying them by name for official records, whether the victims were dead or alive, and took the surviving victims immediately to the hospital. Those who died would be taken to the mortuary later.

“I have to identify quickly who died and who is still alive. This helps to know who needs quick transport to the hospital and who can wait”. (Participant 8)

It was also noted that traffic police officers are responsible for notifying next of kin, either personally, by phone, or through social media, to make it easier for relatives to visit their loved ones at the hospital or to confirm the identity of a deceased relative’s body at the mortuary.

#### Transporting RTI victims to the hospital

The officers reported that transporting RTI victims to the hospital was a crucial responsibility. This responsibility was taken as a priority regardless of the condition of the injured person; private cars and sometime police patrol cars were mentioned as means of transport used to take victims to the hospital. Police officers reported using different approaches, including begging Good Samaritans who owned a car to take victims to hospitals, or forcing a private car owner to take victims to the hospital, especially when nobody agreed to help.

“Most times, drivers do not want to carry injured victims at all, especially if an injured person is profusely bleeding. That is why we normally apply a little bit of force”. (Participant 5)

Additionally, some participants reported using their own money to pay a taxi driver in order to take a victim to the hospital, especially if others refused to do so.

“I always hate begging behaviour. This is because you might ask for help in the first car and the driver comes up with a lot of excuses.... In that case I take my own money, hire a taxi, and ask the driver to rush the victims to the hospital”. (Participant 9)

### Theme 2: overwhelmed while working with limited resources and support

Participants reported experiencing challenges in providing care to injured victims. These challenges were related to resources, infrastructure, and lack of support.

#### Working with scarce resources for helping victims

Participants claimed to have no first-aid kit or other first-aid materials like stretchers in the police cars or at the police posts that could be used for initial care. The officers reported using victims’ or bystanders’ clothes to prevent direct blood contact when helping victims. Others reported using plastic bags as protection against direct blood contact, especially in the presence of profuse bleeding. Although this method was mentioned by several participants, some declared that the method was not effective and that it did little to prevent cross-infection. Some participants reported helping bleeding victims with bare hands due to a lack of protective gear, which created the possibility of exposure to blood-borne infections such as HIV and hepatitis.

“We are using plastic bags; however, they don’t even fit the hand and you find you are busy caring for a victim and the plastic bag has dropped off or even if it is there, you have already contacted the victim’s blood; we are really working in a difficult environment, and sometime it’s even a shame to tell a person that we use plastic bags”. (Participant 2)

#### Difficulty facilitating victims’access to health facilities

Concern was expressed that even if a traffic police officer arrived early at the scene, the injured victim would still be unable to get to the hospital quickly. The situation is even worse if a crash happens at night because many motorists do not even stop when police officers try to pull them over, and if they stop, most of them do not agree to carry injured victims, especially those who are bleeding. Badly potholed roads, sometimes filled with water, were also mentioned as a cause of delay in getting an RTI victim to the hospital.

“There is no system which enables an ambulance to take injured people from the scene, though from what I believe this is what it supposed to happen; taking injured people from the scene to the hospital … It’s really painful, whereby you have toiled enough using other means to make sure the injured person reaches the hospital, just to find that there are three ambulances parked at the hospital. This is unfair and a misuse of resources”. (Participant 3)

#### Overwhelmed and exhausted by responsibilities

Some participants, especially those with high rank in their working area, reported having a lot of responsibility, which overwhelmed them and kept them from having any resting time at all. It was noted that the workload increased when a crash happened because during that time traffic police officers were required to continue fulfilling their legal responsibilities while dealing with newly emerged caring and transporting responsibilities.

“When I receive a call about an accident, that is where the stress starts exactly … . It reminds me of other accidents that I don’t even want to remember at all. It also increases stress because I know that even after my arrival, I have nothing to do apart from taking them to hospital”. (Participant 7)

Participants further reported feeling demoralized because they received so little recognition for their efforts to help RTI victims, especially from bystanders who ended up blaming the traffic police officer for not arriving at the scene on time and not delivering care at the scene the way they thought it should be done.

#### Lack of a support system at the scene and at health facilities

The officers related that once a crash occurs, bystanders crowd the scene without offering any assistance to the police in helping the victims. Instead, they take photos of the victims or steal their property Another challenge the officers reported was the experience of arriving with victims at the hospital and waiting a long time before care was given.

“I don’t know what is wrong with our hospital’s admission system. It’s normal to arrive with an injured victim and you are asked to go and open a patient file and do all the other procedures which are required for admission. This can take up to three hours, and I consider it as a waste of time with no reason because that is not my work. I was just a rescuer”. (FGD 3, Participant 2)

### Theme 3: improving the support system and empowering frontline personnel

Participants expressed their views on strategies that could be employed to address challenges encountered as they provide initial post-crash care to injured victims.

#### Strengthening the emergency care system

Almost all participants expressed the need to have an established emergency care system in Tanzania. Moreover, they suggested having ambulances parked in hot spots along main roads where crashes commonly occur. Participants also suggested that once such an emergency care system was in place, both traffic police and community members around hot spots should be given an emergency telephone number to call in case of a crash or any other road emergency. It was proposed that all hospitals have a special number, as the police do, that would be known by community members and used to call for help when an emergency occurred or to request an ambulance from a nearby health facility. Moreover, participants suggested that having good communication between the on-scene rescuer and the hospital would help hospital staff prepare for emergency arrivals and reduce unnecessary delays.

“Hospitals should have an emergency number to call as one of their services, as police do.... Anybody who has an emergency should be encouraged to call that number, and that will help to release ambulances stationed in the hospital to the scene and save lives”. (Participant, 1)

#### Availability of resources and emergency medical support system

The participants proposed that first-aid materials be distributed to their police posts and their patrol cars. They argued that if first-aid materials could become available, they could even ask bystanders to help with rescuing injured victims, as opposed to what is currently happening, because no one is ready to work without protection. It was also noted that, as police patrol cars are few and ambulances are not in place, increasing the number of patrol cars would mitigate the problem of transporting victims to the hospital. They also suggested that once the number of police patrol cars was increased, a few could be specifically identified and reserved for transporting victims. The need to employ more police officers who would be trained in first aid and who would be specifically available for caring for injured victims was also expressed.

“ Our patrol cars need to be specified, which one is for injured victims and which one should do the rest of activities … . I remember one day we arrested a person with illegal dirty car oil and we put them in the patrol car and when we were on our way to the police station, we found an accident and we had no way other than to take the injured victim in an oil-dirty car”. (Participant 10)

#### Training for police and drivers on victims’ first-aid care and road safety

First-aid training would help both police and drivers, especially bus and taxi drivers, deliver better care to RTI victims. The officers mentioned training as an important issue because most of them declared that when they arrived at a crash scene, their main role was to facilitate transport of the victim to the hospital. This often-meant recruiting taxi drivers or using their own patrols cars; in either case, little or no first aid could be given to the victim. Based on the expression of the need for training, suggestions for workshops on how to initiate care of ill and injured victims were highlighted. Participants were of the view that such training would improve how they provided care and reduce needless fatalities. Furthermore, most of them reported being influenced by the command-driven nature of their work, which is opposite to what care need. Finally, participants pointed out that some drivers ignored road rules and regulations, causing crashes. Therefore, they suggested mandatory refresher courses on road rules and regulations as a way to reduce the number of RTIs. Some police officers liked the idea of the proposed training, while others were against it, as they perceived post-crash care as an extra activity that was not their responsibility.

“You know everybody has specialized in a certain work. My work is not to care for injured victims, you [the interviewer] are the people who are trained about that. Asking me to initiate care to injured victim is like increasing extra work to me, but I have no way out”. (FDG 3, Participant 10)

## Discussion

We have explored the experiences of traffic police officers in regard to the provision of care to RTI victims. These findings provide useful insights into post-crash care provided by this group of responders, as well as areas that need improvements in Tanzania’s emergency care system. On the basis of our findings, we recommend that the following issues relating to traffic police post-crash care of RTI victims be given special attention:

- Maintenance of safety during provision of post-crash care at the scene and on the way to the hospital
- Assurance of availability of resources and support for optimal post-crash care
- Improvement of the post-crash care support system and empowerment of frontline personnel

### Maintenance of safety during provision of post-crash Care at the Scene and on the way to the hospital

Prehospital care differs worldwide in term of level and provider’s knowledge and skills. For countries where an EMS is not in place, the advice is to build it via the identified first responders [12, 27]. Regardless of level, studies show that an EMS is an important service that can save injured victims’ lives and reduce disability [28, 29]. In countries where an EMS is in place, the rate of RTI deaths is 25% lower than in countries without EMS [30]. This is because provision of post-crash care via an EMS is organized, timely, and safe, and its delivery is done by trained personnel using well-equipped ambulance for transporting victims [30, 31].

In the present study, traffic police officers reported that one of their responsibilities after arriving at the scene was to provide some initial injury management and other aid to victims and facilitate their transport to the hospital. Although this was considered “an initial care to injured victims,” the study found that the concept of first aid is not well understood by traffic police and that for them, the provision of first aid basically involves interventions such as extricating victims from the scene and transporting them to the hospital for further management. However, it was not clear from the study how the traffic police ensured initial forms of management of injured victims such as bleeding control, neck immobilization, and splinting of fractured bones measures that optimize outcomes. Providing initial help without applying such safety principles could expose victims to further injury or result in permanent disability or even a decreasing their survival time. This could be why victims who are cared for at the scene and/or transported by police to the hospital are more likely to die than those attended by emergency medical personnel or other first responders [21, 32]. Describing the initial interventions without mentioning application of safety principles, as was the case in many interviews in the present study, indicates a lack of proper knowledge and skills for providing care to RTI victims on the part of traffic police. Poor knowledge and skills in regard to the provision of care to RTI victims by first responders has been demonstrated in other studies to be the main reason for inappropriate intervention during care provision [22, 33]. This situation calls for immediate remedies; otherwise, the police officers who are first at the scene will continue to be of no help to victims, except to rush them to the hospital with poor outcomes afterward [34].

Several studies done elsewhere reveal that most crash scene care providers perform no intervention at all, or even impede the provision of proper care due to limited knowledge and skills [35]. Working with limited knowledge during lifesaving time not only decreases chances of survival for injured victims but also increases the risk of a care provider becoming a victim immediately or in the near future [36]. Most officers are aware of the risks associated with care provision to RTI victims; therefore, they tend to fear helping victims, hence hinder care provision. It has been suggested that proper handling of the victim increases confidence that one will perform well in a subsequent similar event [37].

### Assurance of Availability of resources and support for optimal post-crash care

Traffic police work has been reported to be annoying, and even to be one of the most challenging jobs in the world [38]. The situation gets worse when traffic officers are attending a crash scene with limited or no resources for care provision to injured victims [39]. Despite not having appropriate equipment for care provision, they are required to provide care as its within their job description, and is stipulated as part of their responsibilities. It is not well planned, because the police officers are left in in a dilemma: to take the risk and try to save, or not try to save. Therefore, officers are forced to save for humanitarian reasons as well as to fulfil their obligations, by improvising available materials like using personal cars for transport, plastic bags as gloves, and clothes in order to avoid direct blood contact. Unavailable equipment and supplies not only limits officers’ ability to provide quality care to RTI victims, but also puts them at risk of suffering biological, physical, chemical, and even psychosocial problems [36].. Moreover, regardless of whether or not care is provided at the scene, transporting victims to the hospital has been reported to be an issue because of inaccessible ambulances. The unavailability of ambulances to evacuate RTI victims leads police officers to commandeer private cars to evacuate victims from the scene to the hospital. Such vehicles are not designed to carry victims, and in case of spinal cord injury, further damage to the spine is more or less a guarantee. The situation of not having ambulances for victim evacuation was also found in many other African countries [27]. Those people who are forced to convey victims to the hospital in their private cars do not have knowledge about transporting and caring for injury victims; furthermore, their cars are not designed to carry these victims [17, 34]. When inappropriate vehicles driven by unauthorized and/or ill-qualified persons are used to transport victims to the hospital, the probability of getting there safely is low [21].

Additionally, it was not good to hear from the officers interviewed for the present study that their efforts were sometimes for naught because of delays in the hospital admission process.

Poor infrastructure, especially bad roads, was mentioned as another challenge officer encountered when transporting victims to hospitals. Potholes and other forms of roadway damage not only slow transport, they also increase the likelihood of further injury because victims are not properly secured in the car. These findings align with a systematic review study on LMICs that showed that challenges that the confront prehospital providers at the scene are lack of ambulances, poor infrastructure, poor communication and coordination, and unavailable equipment and personnel [40].

Most of the challenges mentioned by the officers could be reduced or eradicated by coordination between government, prehospital providers, and hospitals. It is important to recognize that these challenges can be tackled by either short- or long-term planning. The government should make sure that a conducive environment is created for the provision of post-crash care by traffic officers and other identified prehospital providers [41, 42]. This would consist of developing, funding, implementing, and enforcing policies to enhance provision of the best possible care at the crash scene. Moreover, the EMS should be strengthened through effective coordination of personnel, resources, and activities, as well as clear delineation of the roles and responsibilities of the team, with effective mechanisms of accountability throughout.

### Improvement of the post-crash care support system and empowerment of frontline personnel

Lack of training in prehospital care for first responders has been identified as a barrier to the provision of prehospital care [40]. To overcome this barrier, formal training in prehospital care should be impacted to officers to establish EMS [12] and meet health system standards and regulations which many LMIC countries are yet to establish [27]. The focus of training should be on basic lifesaving skills; such training is cheap and easy to implement [31]. The training should not be limited to traffic officers; other identified prehospital providers, such as taxi drivers, should also be considered for training [43]. The training has been shown to be cost-effective: Similar training has been done in other countries, and participants have shown retention of skills and knowledge to a significant level [44], with improved patient outcomes the result [28].

Improvement of infrastructure will hasten the arrival of the rescuer to the crash scene and reduce the time spent getting to the hospital. Improved infrastructure will also increase the preparedness of hospital providers, with quicker reaction times saving lives. Lastly, medical care organizations can better satisfy their responsibility to save lives by placing care providers closer to potential crash scenes and increasing collaboration with the police officers who have been identified as, in many cases, the first responders.

## Implications of the study

We explored experience of police officers in provision of initial care to RTI victims at the scene. Findings of this study revealed the important role of police officers in providing care to RTI victims at the scene. For effective outcome of post-crash care, such care needs to be appropriate, timely and safe. To equip police officers better for that role, lifesaving skills and basic principle of post-crash care should be an inclusive topic in police training curriculum and one of the competencies to be acquired during their initial training. To ensure Traffic Police Officers effectively fulfil this role, the government, through the Ministry of Internal Affairs in collaboration with Ministry of Health, should provide police with necessary equipment that can be utilized in provision of basic post-crash care to RTI victims at the scene. To facilitate easy access to formal hospital care and ensure maximum outcome to RTI victims, these findings suggest the need for government efforts of establishing an organized EMS that ensure safe and appropriate continuum of care from the scene to the hospital.

## Limitations of the study

We explored traffic police experiences of providing post-crash care, trying to understand officers’ role, challenges, and opinions about improvements. We acknowledge that similar insights can be acquired from taxi drivers, truck drivers, and community members, who have been found to be the main first responders in other studies [34, 44]. In future studies, it will be interesting to capture their perspectives and contrast them with those of the traffic police who, apart from being involved in providing initial care directly to victims, have legal responsibilities at the scene. It is also important to know how traffic police officers perceive their caring role and the challenges to optimal post-crash care, as they are also involved in direct initial care and play a key role in supporting victims’ access to health facilities.

Ten interviews and three FGDs were conducted for the present study, which might seem a small sample. However, during the last interviews, the same issues emerged repeatedly, and the variety of informants from a range of educational and police ranks allowed us to gather information derived from diverse perspectives and experiences.

This study triangulated method by in-depth interviews and focus group discussion to enhance credibility. Interviews were conducted in Swahili and then translated to English; we acknowledge that some of the meanings of the participants might have been lost in the translation process. However, two authors with Swahili as their native language were involved in the translation process.

## Trustworthiness

When the study findings are worth believing, the study is said to be trustworthy [45]. In a qualitative study, trustworthiness is assessed on the basis of four criteria: credibility, transferability, dependability, and confirmability [46]. In the present study, credibility was ensured through triangulation of participants with various experiences who shed light on the research question from a variety of aspects. To enhance credibility and dependability, the data collected from individual interviews were triangulated with those from FGDs during the analysis process [47–50], and selected codes, categories, and themes were shared among the co-authors, who gave critical comments and suggestions based in large part on their diverse backgrounds and degrees of familiarity with the setting. To confirm that the findings reflected the informants’ experiences rather than the researchers’ understanding of the problem, the presented findings were supported by codes and quotes. Transferability was enhanced by describing the study context and the processes for data collection and analysis in order to show that the results can be used in other contexts and among other traffic police officers.

## Conclusion

Traffic police are key post-crash care providers for RTI victims. However, lack of appropriate lifesaving knowledge and skills among traffic police results provision of unsafe interventions, minimizing the chances of surviving of RTI victims. Working with scarce resources and being overwhelmed by other legal responsibilities keep traffic police officers who serve RTI victims from providing optimal care. A lack of specified means of transport for injured victims delays timely access to health facilities, increasing the chances of death and disabilities resulting from delayed interventions. For optimal post-crash care delivery, efforts should be made to improve the post-crash care support system and empower frontline personnel, including traffic police. Government and other relevant authorities should ensure that traffic police and other first responders are well trained in basic skills for delivery of post-crash care at the scene. Furthermore, the government should invest in establishing a trauma prehospital care system, which would consist of accessible, available, and properly equipped ambulances and trained traffic personnel. However, more research will be needed to determine the acceptability and efficacy of such a system in the Tanzanian context.

## Data Availability

The data sets generated and/or analysed during the present study are not publicly available due to security reasons, as they involve data on individual police officers, but are available from the corresponding author on reasonable request.

## Abbreviations

ALS: Advance life support
BLS: Basic life support
EMS: Emergency medical system
FGD: Focus group discussion
GDP: Gross domestic product
HIC: High-income country
IGP: Inspector General of Police
LMICs: Low- and middle-income countries
MUHAS: Muhimbili University of Health and Allied Sciences
RTI: Road traffic injury
WHO: World Health Organization
